# Poly(Thiophene)/Graphene Oxide-Modified Electrodes for Amperometric Glucose Biosensing

**DOI:** 10.3390/nano12162840

**Published:** 2022-08-18

**Authors:** Maria I. Pilo, Sylwia Baluta, Anna C. Loria, Gavino Sanna, Nadia Spano

**Affiliations:** 1 Dipartimento di Scienze Chimiche, Fisiche, Matematiche e Naturali, Università di Sassari, Via Vienna 2, 07100 Sassari, Italy; 2 Faculty of Chemistry, Wrocław University of Science and Technology, Wybrzeże Wyspiańskiego 27, 50-370 Wrocław, Poland

**Keywords:** amperometric biosensor, conducting polymers, poly(thiophene), glucose oxidase, graphene oxide

## Abstract

The availability of fast and non-expensive analytical methods for the determination of widespread interest analytes such as glucose is an object of large relevance; this is so not only in the field of analytical chemistry, but also in medicinal and in food chemistry. In this context, electrochemical biosensors have been proposed in different arrangements, according to the mode of electron transfer between the bioreceptor and the electrode. An efficient immobilization of an enzyme on the electrode surface is essential to assure satisfactory analytical performances of the biosensor in terms of sensitivity, limit of detection, selectivity, and linear range of employment. Here, we report the use of a thiophene monomer, (2,5-di(2-thienyl)thieno [3,2-b]thiophene (dTT-bT), as a precursor of an electrogenerated conducting film to immobilize the glucose oxidase (GOx) enzyme on Pt, glassy carbon (GC), and Au electrode surfaces. In addition, the polymer film electrochemically synthetized on a glassy carbon electrode was modified with graphene oxide before the deposition of GOx; the analytical performances of both the arrangements (without and with graphene oxide) in the glucose detection were compared. The biosensor containing graphene oxide showed satisfactory values of linear dynamic range (1.0–10 mM), limit of detection (0.036 mM), and sensitivity (9.4 µA mM^−1^ cm^−2^). Finally, it was tested in the determination of glucose in fruit juices; the interference from fructose, saccharose, and ascorbic acid was evaluated.

## 1. Introduction

Glucose detection is an object of great attention by the scientific community due to its relevance in clinical medicine, as well as in the food and beverage industry. Its level in the human blood is indeed related to some diseases, mainly diabetes and obesity [1,2,3,4,5,6]. Therefore, rapid and accurate methods to evaluate its concentration in foods as sources of glucose are widely investigated. The constant interest towards glucose determination is attested by the high number of scientific articles published in the last two decades [2,3,5,7,8]. Conventional analytical methods can be successfully adopted in glucose determination, including chromatographic, spectroscopic, and electrochemical techniques [9]. In particular, electrochemical sensing of glucose is widely utilized because of its high sensitivity and selectivity, accuracy, fast responses, low cost, and ease of use. Electrochemical sensing of glucose can be achieved by an enzymatic or a non-enzymatic approach. The second one is often affected by a relatively low selectivity compared to the enzymatic approach, where the selectivity is assured by the biological receptor (glucose oxidase enzyme, GOx) as a catalyst for the glucose oxidation.

From a general point of view, amperometric enzyme-based sensors can be classified into three groups, according to the nature of the redox mediator used to regenerate the active form of the enzyme (first- and second-generation biosensors), or by the lack of mediator (third-generation biosensors) [10]. Especially, first-generation amperometric glucose biosensors use O_2_ as an electron acceptor to regenerate the active (oxidized) form of the enzyme after the reaction with the substrate (glucose); moreover, the amperometric measurement of the depleted O_2_ or of the formed H_2_O_2_ is directly related to the concentration of glucose. Reduced sensitivity and an upper limit of linearity, as well as a significative effect of interferent substances, are the drawbacks of these sensors; various attempts have been proposed to overcome them. For instance, permselective films and (carbon-based or metallic) nanomaterials can be used to improve the selectivity of the sensor [11,12,13,14]. However, the response of first-generation biosensors is highly influenced by the variation of oxygen concentration in solution; thus, the use of alternative mediators has been developed in second-generation biosensors. In this case, a so-called artificial electron acceptor is employed, dissolving it in the analyte solution or locking it with the enzyme on the electrode surface; furthermore, the amperometric signal produced in the re-oxidation of the mediator is used as a measure of the glucose concentration. Quinone and ferrocene derivatives, ferricyanide, transition-metal complexes, organic conducting salts, and redox-conducting polymers can be used as a mediator in second-generation biosensors [15,16,17,18]. The performances of these kinds of sensors depend on the effectiveness of the communication between the enzyme and the mediator. In this context, immobilizing the mediator to the enzyme could be a better choice than adding it to the analyte solution. However, the mutual immobilization between enzyme and mediator influences the movement of the enzyme and its interaction with the substrate; thus, this limits the efficiency of the measure. Therefore, the use of redox mediators in solution is still adopted in the design of second-generation biosensors. The possibility of assuring a direct electron transfer between the enzyme and the electrode, avoiding the need of a redox mediator, is finally given by the so-called third-generation biosensors. Their performances strongly depend on the distance between the biological receptor and the electrode surface; such a requirement represents the main trouble in their construction.

In any case, one of the most critical steps in the development of an electrochemical biosensor is the efficient and long-term immobilization of the enzyme on the electrode surface. Different approaches are possible with this aim, including physical absorption, entrapment in gels or membranes, and covalent- or cross-linking [19,20,21]. In this context, conducting polymers are extensively used as immobilizing agent for the biological receptor, with two main, different strategies. As a first approach, the enzyme is dissolved in the solution containing the monomer; then, it is entrapped in the conducting film during its electrochemical synthesis. This is an efficient approach; however, it requires quite a high concentration of the enzyme and is quite expensive. Furthermore, the conditions suitable for the activity of the enzyme often do not fit with the electropolymerization requirements; for instance, as for the nature of the solvent or pH value. As an alternative, a double-step strategy can be adopted involving, first, the electro-synthesis of a conducting polymer film on the electrode surface; then, the (physical or chemical) immobilization of the enzyme on the film surface. Especially, thiophene-based electrochemically generated conducting polymers have been proposed as components of electrochemical biosensors; this is due to their high conductivity and stability [19,22,23,24,25,26,27,28,29,30]. Most polythiophenes can be obtained by electrochemical synthesis in non-aqueous solvents; thus, the double-step approach is often adopted in this case.

In order to improve the anchoring of the bioreceptor on the film surface, as well as the electron-transfer rate of the biosensors, nanomaterials can be used alone or in addition to other components of the biosensor. Among nanomaterials, graphene oxide has been successfully used due to its good electric conductivity, large surface area, and excellent mechanical properties [31,32,33,34].

**Figure 1:**
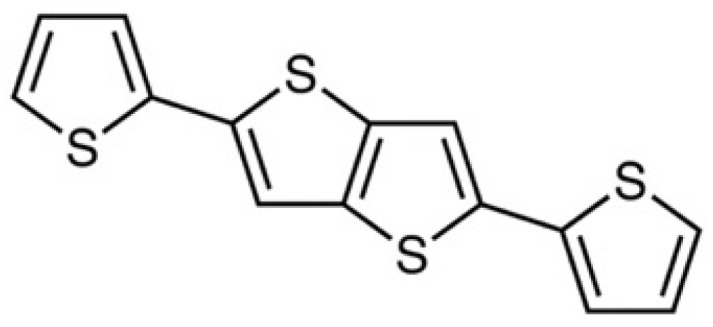
Structure of 2,5-di(2-thienyl)thieno[3,2-b]thiophene (dTT-bT).

Looking at the briefly described features above, second-generation biosensors using a redox mediator in the substrate solution can be regarded as a good compromise between ease of assembly and analytical performances in terms of sensitivity, accuracy, and selectivity. However, highly fascinating designs of electrochemical biosensors reported in the scientific literature often require an expensive and time-consuming approach aimed at immobilizing glucose oxidase and at excluding interferences; they involve one or more preliminary synthetic steps. Therefore, our purpose is to propose as an alternative, GOx-based biosensors obtained from low-cost, commercially available starting materials that are easy and quick to prepare, and suitable for fast and accurate glucose determinations in commercial beverages. With this aim, here we report the use of a thiophene derivative (2,5-di(2-thienyl)thieno[3,2-b]thiophene, dTT-bT, Figure 1) as a precursor of an electro-generated conducting polymer film; modified with graphene oxide (GrO) in a glucose oxidase-based amperometric biosensor for the detection of glucose in fruit juices. The development of the electrochemical biosensor reported here involves a few simples steps: (i) electrosynthesis of a polythiophene derivative as a transducer on an electrode surface, starting from a commercial, affordable precursor; thus, this avoids time- and money-expensive synthetic steps; (ii) modification of the conducting polymer layer by dropping a methanol suspension of graphene oxide in methanol; and (iii) efficient immobilization of the glucose-oxidase enzyme on the modified electrode by dipping in an aqueous solution containing the enzyme and a condensing agent. The analytical performances in terms of sensitivity, LoD, accuracy, and linear dynamic range, as well as the selectivity against fructose, saccharose, and ascorbic acid, are reported. The behavior of biosensors not containing graphene oxide was also investigated and compared to the polythiophene film/GrO/GOx one.

## 2. Materials and Methods

### 2.1. Reagents and Apparatus

Tetraethylammonium hexafluorophosphate (TEAPF_6_, puriss. electrochemical grade), *p*-benzoquinone (BQ), N-cyclohexyl-N’-(2-morpholinoethyl)carbodiimide metho-p-toluenesulfonate (CMC), graphite oxide powder (GrO), sodium monohydrogen phosphate, potassium dihydrogen phosphate, 𝛽-D-glucose, glucose oxidase (GOx) from *Aspergillus niger*, saccharose, and ascorbic acid were from Sigma-Aldrich (Milan, Italy); CH_2_Cl_2_ (99.8%, packaged under nitrogen) was from Acros; 2,5-di(2-thienyl)thieno[3,2-b]thiophene (dTT-bT) was from TCI Chemicals (Zwijndrecht, Belgium); and the fructose and real samples (pear and apricot juices) were from a local food company. All the buffers were prepared according to generally known, obligatory standards.

A CHI-650 electrochemical station (CH Instruments, Austin, TX, USA) interfaced with a PC using its software was used in all the electrochemical tests. The experiments were performed in a three-electrode, single compartment cell equipped with a Ag/AgCl reference electrode, a graphite bar as an auxiliary electrode, and a Pt (diameter 2 mm), glassy carbon (GC, diameter 3 mm) or Au (diameter 2 mm) disk as a working electrode (WE). Working electrodes were polished with alumina powder (1 and 0.3 µm diameter), treated in an ultrasonic bath for 10 min, and rinsed with water and acetone before use in polymer film deposition.

### 2.2. Biosensor Preparation

Electrochemical polymerization of dTT-bT was performed in a 5 mL CH_2_Cl_2_ solution containing 0.1 M TEAPF_6_ as a supporting electrolyte and 1 mM monomer, purging Ar gas into the cell for 20 min before each experiment. A polymer film was formed on the electrode surface (Pt, GC, or Au disk) by applying a potential value between 0.98 V and 1.05 V; this was according to the cyclic voltammetry characterization on the different electrode materials, until a charge of 1 mC was passed. The film was neutralized by applying a potential of 0 V for 60 s; then, characterized by cyclic voltammetry in a CH_2_Cl_2_ solution containing only 0.1 M TEAPF_6_; and finally, washed with ultrapure water.

A first set of biosensors (WE/poly(dTT-bT)/GOx) was obtained via immobilizing the GOx enzyme by dipping the film in an ultrapure water solution (2.5 mL) containing CMC (7.5 mg) and GOx (30 mg) at 4 °C for at least 12 h [22].

A second set of biosensors (WE/poly(dTT-bT)/GrO/GOx) was realized via modifying the film through the dropping of a suspension of GrO in methanol (1 mg/1 mL) on the polymer layer; then, letting the solvent evaporate at room temperature. The GOx enzyme was finally immobilized on the polymer/GrO surface as described for the first set.

### 2.3. Glucose Sensing

The WE/poly(dTT-bT)/GOx and WE/poly(dTTbT)/GrO/GOx biosensors were used as a working electrode in a three-electrode cell containing 20 mL of a phosphate buffer solution (0.1 M, pH 7.0) with a 1 mM BQ as a redox mediator. A constant potential of 0.40 V vs. Ag/AgCl was applied to the stirring solution, and the current was measured as a function of the time. When the background current was stabilized, incremental additions of a 0.2 M 𝛽-D-glucose aqueous solution were conducted; this allowed the signal to stabilize between two subsequent additions (usually 200 s).

The same conditions were adopted to test the poly(dTTbT)/GrO/GOx biosensor on real samples. Pear and apricot juices were properly diluted as 1:100 in 20 mL of the 0.1 M phosphate buffer solution (containing 1 mM BQ) at pH = 7.0; they were analyzed for glucose without further treatment. The glucose concentration was estimated by the standard addition method.

In all cases, the sensitivity was calculated as the ratio of the slope of the calibration curves to the area of the electrode surface, while LoD (limit of detection) was calculated as:

LoD = 3.29 σ_B_/b (1)

where σ_B_ is the standard deviation of the blank (calculated on 11 replicates of the blank) and b is the slope of the regression line [35].

All the analytical measurements were performed in triplicate. For the sake of simplicity, one representative image for each analytical experiment is reported through the text.

## 3. Results

### 3.1. Electrochemical Investigation of dTT-bT

**Figure 2:**
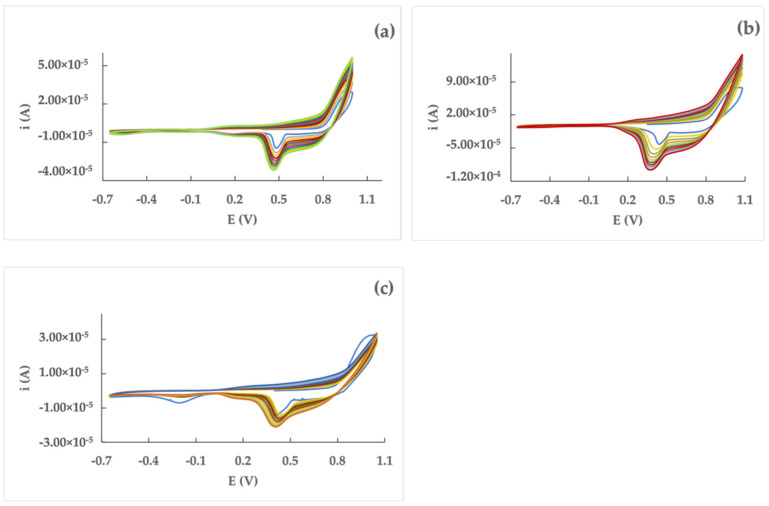
Cyclic voltammetry polymerization (10 cycles) of 1 mM dTT-bT in 0.1 M TEAPF_6_/CH_2_Cl_2_ solution. WE: (**a**) Pt; (**b**) GC; (**c**) Au. RE: Ag/AgCl. CE: graphite bar. Potential scan rate: 100 mV s^−1^.

The electrochemical characterization of 2,5-di(2-thienyl)thieno[3,2-b]thiophene (dTT-bT) was performed using a Pt, GC, or Au disk as a working electrode. In all cases, the cyclic voltammetry behavior evidenced an anodic response at 0.99 V, 1.05 V, and 0.98 V, respectively; there was a sharp peak (0.48 V, 0.42 V, 0.41 V) in the reverse scan. In all cases, subsequently scanning the potential in the range suggested by the voltammetric responses indicated that the peak current increased (Figure 2); in addition, the presence of a red-orange film was observed on the electrode surfaces.

**Figure 3:**
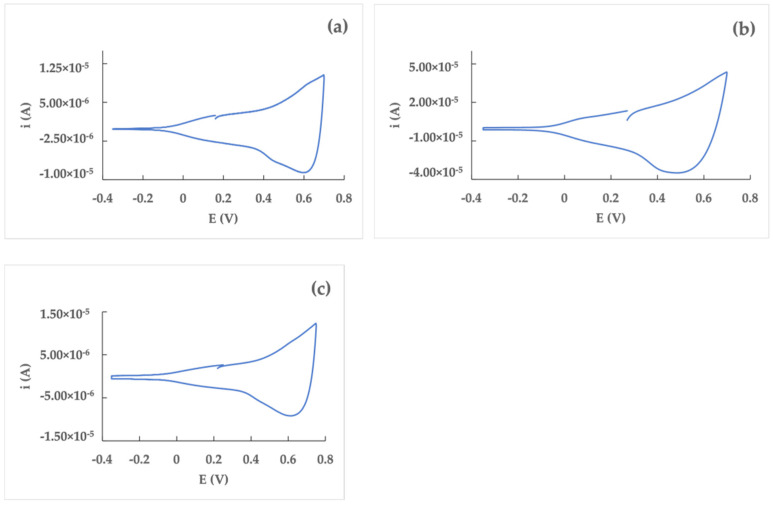
Cyclic voltammetry characterization of poly(dTT-bT) on the Pt (**a**), GC (**b**), and Au (**c**) electrode surfaces. 0.1 M TEAPF_6_/CH_2_Cl_2_ solvent system. Potential scan rate: 100 mV s^−1^.

The films grown by potentiodynamic polymerization on each electrode surface were characterized by cyclic voltammetry in a monomer-free 0.1 M TEAPF_6_/CH_2_Cl_2_ solution. A doping process was always observed in the range −0.35 ÷ 0.7 V, with an associated de-doping process between 0.5 V and 0.6 V (Figure 3).

In order to better tune the features of the films, the electrochemical polymerization of the monomer was performed by chronoamperometry, applying a constant potential (selected according to the voltammetric characterization) to the working electrode until a charge of 1 mC was attained. The voltammetric characterization of the potentiostatic-grown films confirmed their doping–dedoping behavior as for the potentiodynamic-grown films; the current values were lower in the potentiostatic-mode polymers due to their lower thickness compared to the potentiodynamic-mode ones.

### 3.2. Glucose Sensing

#### 3.2.1. WE/Poly(dTT-bT)/GOx Sensors

**Figure 4:**
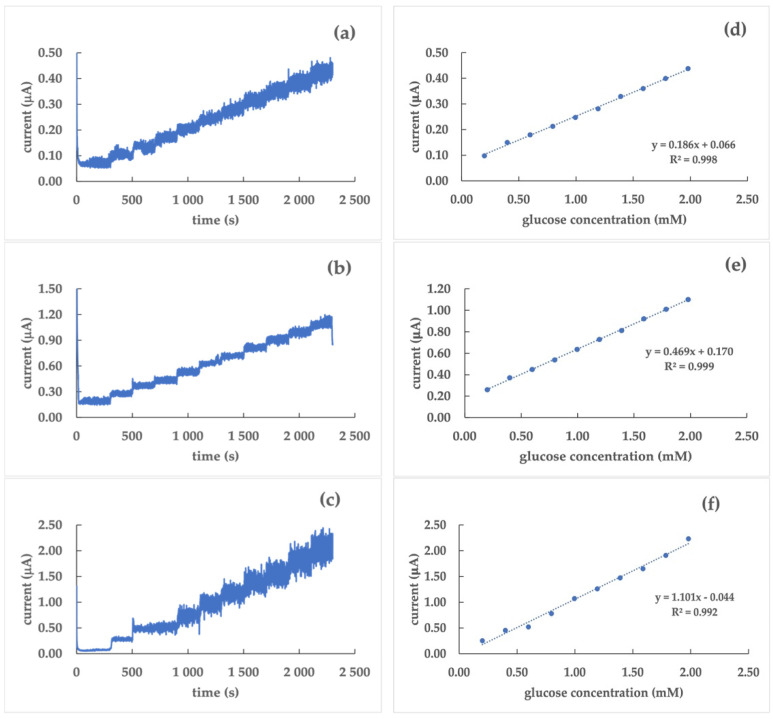
Current/time responses (**a**–**c**) and corresponding calibration curves (**d**–**f**) of Pt/poly(dTT-bT)/GOx (**a**,**d**), GC/poly(dTT-bT)/GOx (**b**,**e**), and Au/poly(dTT-bT)/GOx (**c**,**f**) biosensors in a phosphate buffer (0.1 M, pH 7.0) at a glucose concentration range of 0.2 ÷ 2.0 mM; working potential: 0.40 V vs. Ag/AgCl.

The poly(dTT-bT) films grown on the Pt, GC, and Au electrode surfaces were used to immobilize the GOx enzyme, as described in Section 2.2. The first set of sensors, namely Pt/poly(dTT-bT)/GOx, GC/poly(dTT-bT)/GOx, and Au/poly(dTT-bT)/GOx, with a film charge equal to 1 mC, was tested towards the determination of glucose in a 0.1 M phosphate buffer at pH = 7.0. The current/time responses (Figure 4a–c) show an acceptable stability of the signal for the sensor using GC (Figure 4b) as an electrode material; whereas the Pt- (Figure 4a) and Au-based (Figure 4c) arrangements show a large noise, increasing with the glucose concentration. All the sensors showed a good linearity between the current and glucose concentration in the range between 0.2 and 2.0 mM (Figure 4d–f), with the higher sensibility (expressed as a slope of the calibration curve) for the Au/poly(dTT-bT)/GOx one.

#### 3.2.2. GC/Poly(dTT-bT)/GrO/GOx Sensor

**Figure 5:**
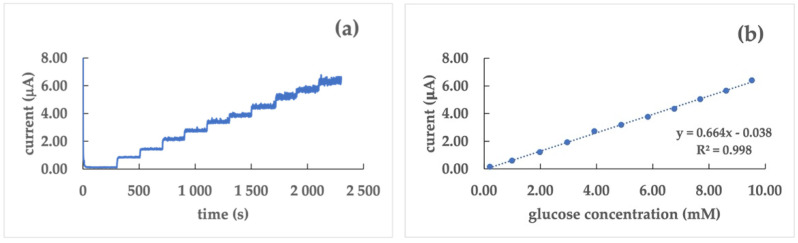
Current/time response (**a**) and calibration curve (**b**) of the GC/poly(dTT-bT)/GrO/GOx biosensor in a phosphate buffer (0.1 M, pH 7.0) at a glucose concentration range of 0.2 ÷ 10 mM; working potential: 0.40 V vs. Ag/AgCl.

GC/poly(dTT-bT)/GrO/GOx sensors were prepared according to Section 2.2. Due to a low stability and repeatability of the current signal evidenced in Pt- and Au-based sensors without and with GrO, only GC was used as an electrode material for the sensors using GrO in addition to the polymer film as a transducer. The comparison between the two GC-based sensors, without and with GrO, suggests that the presence of the graphite powder makes the current/time response (Figure 5a) more stable than that of the sensor without GrO. Furthermore, the calibration curve (Figure 5b) evidences that the presence of GrO into the assembling of the sensor makes it more sensitive; this is indicated by the higher values of the current as well as a higher value of the slope of the calibration curve.

#### 3.2.3. Interferences

**Figure 6:**
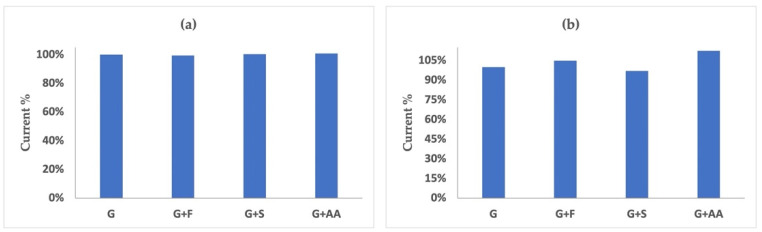
Effect of fructose (F), saccharose (S), and ascorbic acid (AA) on the current value in a 0.2 mM (**a**) and 2.0 mM (**b**) glucose (G) solution; the glucose:interferent ratios are equal to 1:1, 1:0.6, and 1:0.05, respectively.

Interferences tests in the glucose sensing were carried out towards fructose, saccharose, and ascorbic acid with 1:1, 1:0.6, and 1:0.05 glucose:interference ratios, respectively. The effect of all the interfering species was extremely low (RSD% ≤ 0.5) in a solution containing glucose 0.2 mM (Figure 6a). At an increasing glucose concentration of up to 2.0 mM, and an interference concentration accordingly, the effect appears slightly higher; the RSD% is lower than 5% for fructose and saccharose, and lower than 10% for ascorbic acid (Figure 6b).

#### 3.2.4. Real Samples and Recovery Tests

Commercial fruit juice samples (pear and apricot) were employed to measure the content of glucose using the standard addition method. Each experiment was replicated three times. The results are reported in Table 1.

Recovery tests were performed on standard solutions containing 1.0 mM and 5.0 mM glucose concentration. The recovery was calculated as a percentage ratio of the evaluated glucose concentration towards the real value. The recovery results are reported in Table 2.

## 4. Discussion

The voltammetric behavior of 2,5-di(2-thienyl)thieno[3,2-b]thiophene (dTT-bT) was investigated with the aim to find the better conditions for using it as immobilizing agent for a glucose sensor. Tetraethylammonium hexafluorophosphate was selected as a supporting electrolyte based on previous results [22,36,37]; these suggest satisfactory reproducibility and stability of the polythiophene-based conducting polymer films when alkylammonium salts are employed with this role. The voltammetric responses of the monomer on three different electrode surfaces (Pt, GC, and Au), as well as a feature of the following scans, evidence a sharp shape in the backward scans; this suggests absorbing/desorbing phenomena as previously reported for thiophene derivatives [38]. In order to tune the thickness of the film, the electrode surfaces were modified using a chronoamperometric deposition mode. Despite the different dimensions of the three working electrodes (diameter = 2 mm for Pt and Au; 3 mm for the GC electrodes), a charge value of 1 mC was always chosen according to preliminary tests concerning the stability of the film on the electrode surfaces.

The immobilization of the GOx enzyme was first performed directly on the electrogenerated film, to give the WE/poly(dTT-bT)/GOx (WE = Pt, GC, Au) biosensors. By comparing their performances, it is possible to observe the Pt-based sensor has the lowest sensitivity (5.9 ± 0.2 µA mM^−1^ cm^−2^ against 6.6 ± 0.6 µA mM^−1^ cm^−2^ and 35 ± 5 µA mM^−1^ cm^−2^ for GC and Au, respectively), and the higher LoD value (0.91 mM against 0.13 mM for GC and 0.12 mM for Au). On the other hand, the Au-based sensor showed a poorly stable current signal; while the GC-based one allowed the obtaining of a quite stable current signal coupled to acceptable sensitivity and LoD. These results showed that GC as a working electrode showed a higher affinity towards the analyte, which produces the higher and more resolved current peaks; this made the electrochemical analysis easy as compared to other working electrodes. In all cases, the linear dynamic range was from 0.2 mM to 2.0 mM.

For these reasons, only GC was chosen as an electrode surface for the second step, where graphene oxide was dropped on the electrogenerated polymer film before the anchoring of the enzyme. The GC/poly(dTT-bT)/GrO/GOx biosensor shows a higher sensitivity (9.4 ± 0.7 µA mM^−1^ cm^−2^) and a lower LoD (0.036 mM) than the analogue without graphene oxide. Moreover, the current appears more stable compared to the no-GrO arrangement; this allows the extension of the linear dynamic range from 0.2 mM to 10 mM. The improvement of the results for biosensing systems with GrO are connected with its excellent conductive and mechanical properties, as well as a high reactivity to chemical compounds [39]. What is more, the presence on its surface of indigenous “ripples” provide a valuable property in the case of biosensing due to increasing the surface area of the working electrode; this ensures successful immobilization of an enzyme [40].

A further clue on the performances of the GC/film/GrO/GOx arrangement could be obtained by the evaluation of the electrochemical-active surface area [41,42,43] using the Randles–Sevcik equation (Equation (S1)). However, this approach is valid for reversible and diffusion-controlled processes; whereas the behavior of the modified electrode discussed here does not strictly fit such conditions (Figure S1). Hence, only a prudent estimation of the active surface area of GC/poly(dTT-bT)/GrO/GOx can be performed; this suggests that its value is about twice than that of the unmodified electrode.

The effect of the interfering species was tested first against fructose, which is the main simple sugar present in fruit together with glucose. The interference study of fructose towards glucose was performed at a concentration value of 0.2 mM, causing no significative change of current. At higher concentration values (2 mM), a slight increase (+5%) in the current value of the glucose solution due to fructose addition was observed. Analogously, the interference of saccharose was tested on 0.2 mM and 2.0 mM solutions of glucose. In this case, with saccharose being usually present in a lower amount in beverages, its effect was investigated in a 0.6:1 ratio to glucose. Its effect was not significative at the lowest concentration and caused a slight decrease (−3%) of the current value at a higher concentration. Finally, the effect of ascorbic acid, commonly used in the food industry and presenting an oxidation potential rather close to the analyte, was studied (1:0.05 G:AA ratio). As in the case of fructose and saccharose, the presence of ascorbic acid has no significative effect on the detection of glucose at low concentrations (0.2 mM); whereas it causes a 13% overestimation of the current measured in 2 mM glucose solutions. However, it can be pointed out that ascorbic acid concentrations in beverages are usually lower than the values taken into account here; hence, the 13% overestimation reported can be seen as an upper limit value for its interference.

The analytical performances of the WE/poly(dTT-bT)/GOx (WE = Pt, GC, Au) and GC/poly(dTT-bT)/GrO/GOx sensors was compared to some of the GOx-based sensors from the literature (Table 3). Especially in the case of the last one, containing graphene oxide as an interlayer between the conducting film and the enzyme, the analytical data suggest that GrO can induce a short response time (due to a higher surface reactivity) and a high sensitivity (ascribable to an enhanced active surface area). Furthermore, the GrO-containing biosensor shows a behavior making it suitable for the determination in real samples such as human blood, as well as fruit juices. In particular, recovery tests support the use of the proposed biosensor in soft drinks; wherein glucose concentration can be recovered with a high accuracy and with a low level of interferences from fructose and saccharose.

## 5. Conclusions

In this study, the use of a polythiophene conducting film associated to graphene oxide in the construction of a GOx-based, second-generation, amperometric biosensor is reported. The conducting polymer was obtained via potentiostatic polymerization by a commercially available monomer; that is, 2,5-di(2-thienyl)thieno [3,2-b]thiophene. The potentiostatic polymerization approach was selected instead of the potentiodynamic one in order to carefully monitor the thickness of the film through the charge passed during the film growth. The effect of a deposit of graphene oxide on the polymer film before the anchoring of the enzyme was also investigated; an improvement in analytical performances compared to the biosensors obtained in the absence of graphene oxide was observed. The behavior of the GC/polymer/graphene oxide/enzyme proposed sensor towards glucose detection is comparable to (or better than) biosensors previously reported, with the benefit of easily accessible and less expensive starting materials. The design of this last electrochemical biosensor, involving the cooperation of a conducting polymer and graphene oxide, and the presence of *p*-benzoquinone as a redox mediator, also allows a reduction in the effect of potential interferents, such as fructose, saccharose, and ascorbic acid.

## Figures and Tables

**Table 1:** Glucose concentration average values and the standard deviation in the real samples (n = 3 replicates for each sample).

Real Sample	Glucose Concentration Found (M)	Slope (mA/M)	R^2^
Pear juice	0.3 ± 0.1	0.76 ± 0.05	0.99 ± 0.01
Apricot juice	0.40 ± 0.04	0.92 ± 0.03	0.997 ± 0.003

**Table 2:** Recovery tests results (n = 3 replicates).

Glucose Concentration (mM)	Glucose Found (mM)	RSD%	Recovery (%)
1.0	0.95	8.12	94.90
5.0	4.99	0.48	99.80

**Table 3:** Some examples of electrochemical GOx-based sensors for the determination of glucose.

Sensor	Linear Range (mM)	LoD (mM)	Sensitivity (µA mM^−1^ cm^−2^)	Response Time (s)	Stability (Days)	Ref.
Pt/PEDOT/PAA/GOD	0.96–30	0.29	0.59	10–30	30	[21]
Pt/PEDOT/AA/GOD	1.86–30	0.56	0.52	10–30	30	[21]
Pt/poly(2,2′-BT)/GOx	0.09–5.20	0.030	48	180	>15	[22]
Pt/poly(4,4′-BT)/GOx	0.15–5.20	0.050	11	50	>30	[22]
Pt/PPy-GOx/PPy-Cl	0.5–24	0.0269	3.5	3–7	>60	[1]
GC/Py/Py-CO2H/Py-Fc/GOx	1.0–4.0	0.0069	1.796	2	28	[44]
GOx/Pt/rGO/P3ABA/SPCE	0.25–6.00	0.0443	22.01	-	7	[9]
SiO2(LuPc2)PANI(PVIA)-CNB/GOx	1–16	0.1	38.53		45	[45]
Graphite rod/EDOT-PdBPI-co-HKCN/GOx	0.25–2.5	0.176	-	-	>56	[36]
Chit-GOX-pFcAc-HSA-carbon paper	0.1–10	0.07	0.33	200	>28	[15]
Pt/(CHIT/PAA)GOD	0.05–15	0.01	21	<8	60	[46]
Pt/poly(dTT-bT)/GOx	0.2–2.0	0.91	5.9	<200	>30	This work
GC/poly(dTT-bT)/GOx	0.2–2.0	0.12	6.6	<200	>30	This work
Au/poly(dTT-bT)/GOx	0.2–2.0	0.12	35	<200	>30	This work
GC/poly(dTT-bT)/GrO/GOx	0.2–10	0.036	9.4	10–20	>60	This work

PEDOT, poly(3,4-ethylenedioxythiophene); PAA, polyacrylic acid [21]; AA, anthranilic acid; GOD and GOx, glucose oxidase; poly(2,2′-BT), poly(2,2′-bithiophene); poly(4,4′-bis(2-methyl-3-butyn-2-ol)-2,2′-bithiophene); PPy, polypyrrole; Py-CO2H, 1-(2-carboxyethyl)pyrrole; Py-Fc, N-(3-(1H-pyrrol- 1-yl)ethyl)ferrocenecarboxate; rGO, reduced graphene oxide; P3ABA, poly(3-aminobenzoic acid); SPCE, screen-printed carbon electrode; LuPc2, lutetium phthalocyanine; PANI, polyaniline; PVIA, Poly(vinyl alcohol-vinyl acetate) itaconic acid; CNB, conducting nanobeads; EDOT, 3,4-ethylenedioxythiophene; BPI, 1,3-Bis(2-pyridylimino)isoindoline; HKCN, 4-amino-N-(2,5-di(thiophene-2-yl)-1H-pyrrol-1-yl)benzamide; pFcAc, poly(N-(3-dimethyl(ferrocenyl)methylammonium bromide) propyl acrylamide; HAS, human serum albumin; CHIT, chitosan; PAA, poly(allylamine) [46].

## Data Availability

The data used in this work are available from the corresponding author on request.

## Supplementary Materials

The following supporting information can be downloaded at: https://www.mdpi.com/article/10.3390/nano12162840/s1, Figure S1: (a) Cyclic voltammetric curves of GC/poly(dTT-bT)/GrO/GOx in a 0.1 M KCl solution containing 5mM K_3_[Fe(CN)_6_] at scan rates from 0.02 V s^-1^ to 0.2 V s^-1^. (b) Relationship between the peak current and the square root of the potential scan rate.
