# Supplementary material for: Poly(Thiophene)/Graphene Oxide-Modified Electrodes for Amperometric Glucose Biosensing
Source: Nanomaterials (Basel). 2022 Aug 18;12(16):2840. doi: 10.3390/nano12162840 (PMC9413253; doi:10.3390/nano12162840)
Supplement: Supplementary file 1 [file nanomaterials-12-02840-s001.zip › nanomaterials-1863693-supplementary.pdf]

Supporting Material

Poly(Thiophene)/Graphene Oxide-Modified Electrodes for Amperometric Glucose Biosensing

Maria I. Pilo [1,*], Sylwia Baluta [2], Anna C. Loria [1], Gavino Sanna [1] and Nadia Spano [1]

[1] Dipartimento di Scienze Chimiche, Fisiche, Matematiche e Naturali, Università di Sassari, Via Vienna 2, 07100 Sassari, Italy
[2] Faculty of Chemistry, Wrocław University of Science and Technology, Wybrzeże Wyspiańskiego 27, 50-370 Wrocław, Poland
* Correspondence: mpilo@uniss.it

Electrochemically-active surface area

The electrochemically-active surface area can be estimated by the slope of the curve I_p vs $v^{1/2}$ in a 0.1 M KCl solution containing 5 mM $[Fe(CN)_6]^{3-/4-}$ using the Randles-Sevcik Equation (S1):

$$I_p = 2.69 * 10^5\ n^{3/2} A D^{1/2} C v^{1/2} \qquad \text{(S1)}$$

where I_p is the redox peak current in ampere, n is the number of exchanged electrons, A is surface area in cm^2, D is the diffusion coefficient in cm^2 s^{-1}, C is the concentration of electroactive species in mol cm^{-3}, v is the potential scan rate in V s^{-1}.

Assuming n = 1 and D = 6.5×10^{-6} cm^2 s^{-1}, the value of the active surface area for GC/poly(dTT-bT)/GrO/GOx has been estimated equal to 0.083 cm^2, that is about twice than the value estimated for an unmodified GC electrode in the same conditions (0.035 cm^2).

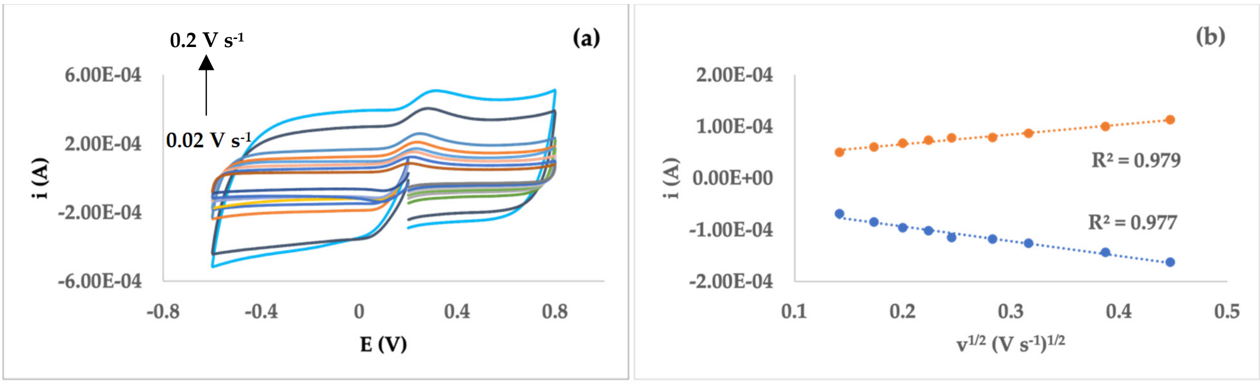

Figure S1. (a) Cyclic voltammetric curves of GC/poly(dTT-bT)/GrO/GOx in a 0.1 M KCl solution containing 5 mM $K_3[Fe(CN)_6]$ at scan rates from 0.02 V s^{-1} to 0.2 V s^{-1}. (b) Relationship between the peak current and the square root of the potential scan rate.
